# Integrative single-cell RNA-seq and ATAC-seq analysis of myogenic differentiation in pig

**DOI:** 10.1186/s12915-023-01519-z

**Published:** 2023-02-01

**Authors:** Shufang Cai, Bin Hu, Xiaoyu Wang, Tongni Liu, Zhuhu Lin, Xian Tong, Rong Xu, Meilin Chen, Tianqi Duo, Qi Zhu, Ziyun Liang, Enru Li, Yaosheng Chen, Jianhao Li, Xiaohong Liu, Delin Mo

**Affiliations:** 1 State Key Laboratory of Biocontrol, School of Life Sciences, Sun Yat-Sen University, Guangzhou, 510006 Guangdong China; 2 Guangdong Key Laboratory of Animal Breeding and Nutrition, State Key Laboratory of Livestock and Poultry Breeding, Institute of Animal Science, Guangdong Academy of Agricultural Sciences, Guangzhou, 510640 Guangdong China; 3 Faculty of Forestry, University of British Columbia, Vancouver, BC V6T 1Z4 Canada

**Keywords:** Pig, Myogenic differentiation, Skeletal muscle, scRNA-seq, scATAC-seq

## Abstract

**Background:**

Skeletal muscle development is a multistep process whose understanding is central in a broad range of fields and applications, from the potential medical value to human society, to its economic value associated with improvement of agricultural animals. Skeletal muscle initiates in the somites, with muscle precursor cells generated in the dermomyotome and dermomyotome-derived myotome before muscle differentiation ensues, a developmentally regulated process that is well characterized in model organisms. However, the regulation of skeletal muscle ontogeny during embryonic development remains poorly defined in farm animals, for instance in pig. Here, we profiled gene expression and chromatin accessibility in developing pig somites and myotomes at single-cell resolution.

**Results:**

We identified myogenic cells and other cell types and constructed a differentiation trajectory of pig skeletal muscle ontogeny. Along this trajectory, the dynamic changes in gene expression and chromatin accessibility coincided with the activities of distinct cell type-specific transcription factors. Some novel genes upregulated along the differentiation trajectory showed higher expression levels in muscular dystrophy mice than that in healthy mice, suggesting their involvement in myogenesis. Integrative analysis of chromatin accessibility, gene expression data, and in vitro experiments identified EGR1 and RHOB as critical regulators of pig embryonic myogenesis.

**Conclusions:**

Collectively, our results enhance our understanding of the molecular and cellular dynamics in pig embryonic myogenesis and offer a high-quality resource for the further study of pig skeletal muscle development and human muscle disease.

**Supplementary Information:**

The online version contains supplementary material available at 10.1186/s12915-023-01519-z.

## Background

Skeletal muscle development is a highly complex and tightly coordinated multistep process [1]. In mammals, all skeletal muscles of the body derive from the somites, which are metameric mesodermal structures that form on both sides of the neural tube. The most dorsal portion of the somite remains epithelial and becomes the dermomyotome [2]. Muscle precursor cells labeled by the paired box transcription factors Pax3 and Pax7 are generated in the dermomyotome and dermomyotome-derived myotome and undergo cell-fate commitment, followed by migration along an established pathway to differentiate into skeletal muscles [3]. In pigs, for example, the development of somites occurs between approximately days 14 and 22 of gestation [4]. Pluripotent mesodermal cells originate in somites and are committed to the myogenic lineage, followed by proliferation of myoblasts and a subsequent two waves of myoblast differentiation and fusion to form primary and secondary myofibers. Primary myofibers are generated at 35–55 days of gestation, followed by the secondary myofibers that form based on the template of the primary myofibers at approximately 50–90 days of gestation [5].

The myogenic process involves complex gene expression regulatory networks, which mainly exert their function through precise regulation of intercellular signaling and the control of specific gene expression. Pax3 and Pax7 are essential upstream regulators of myogenesis. Pax3/Pax7-positive cells in the dermomyotome provide the self-renewing reserve cell population for muscle formation [6]. Myogenic regulatory factors (MRFs, including Myf5, MyoD1, myogenin, and MRF4), as members of the basic helix–loop–helix family of transcription factors, have well known roles in controlling the determination and differentiation of skeletal myogenic cells during embryonic and postnatal myogenesis [7, 8]. In vivo, molecular and genetic experiments in mice, Drosophila, and chickens have uncovered the genetic and epigenetic regulatory mechanisms necessary for skeletal muscle formation [9, 10]. In vitro, the precise process of myogenesis has been extensively studied using the murine immortalized C2C12 myoblast cell line as a model system because of its high proliferation and differentiation capacities [11, 12]. However, similar progress in pig myogenesis has been limited by the lack of a convenient model system and the high heterogeneity of pig primary myogenic cells.

The skeletal muscle of agricultural animals (typically pig, cattle, and beef) is one of the most significant dietary protein sources for human consumption. The development and growth of skeletal muscle determine meat yield and quality [13]. Thus, a better understanding of porcine skeletal muscle development is needed because of the agricultural importance of pigs and increasing awareness of the benefits of using pigs as model organisms for human development and disease [14, 15]. Tibetan pig, an indigenous pig breed of China, used to live only in the plateau, but now they can also live normally in the plains [16, 17]. The meat yield of Tibetan pig is low, but the meat yield is significantly improved in its hybrid offspring with Duroc, with a higher preslaughter weight (49.50 ± 4.23 kg) and a larger loin eye area (33.00 ± 3.84 cm^2^) than those of Tibetan pig (preslaughter weight, 38.00 ± 2.89 kg; loin eye area, 16.83 ± 1.81 cm^2^) [18]. Therefore, using Tibetan pig (ZZ) and Duroc×Tibetan pig (DZ) as models to study the regulatory mechanism of skeletal muscle development would be very beneficial for improving pig farming. Whether there are differences in the early embryonic myogenesis of ZZ and DZ, with the same female parent and different male parent, remains to be studied.

Single-cell RNA sequencing (scRNA-seq) allows the comprehensively profiling of the gene expression changes observed in development at a cellular level [19], while single-cell transposase-accessible chromatin sequencing (scATAC-seq) allows the analysis of chromatin accessibility and transcription factor (TF) binding to be profiled at a similar resolution [20]. Recently, scRNA-seq has been applied to studying cellular heterogeneity in skeletal muscle tissues [21, 22]. Scientific researchers outlined the major mononuclear cell types present in mature skeletal muscle of mouse, ranging from 10 to 15 cell types depending on cluster assignments and the granularity of subtyping [21–23]. The major cell types always include the following broad categories: fibro/adipogenic progenitors (FAPs), tenocytes, endothelial cells, smooth muscle cells, immune cells (B cells, T cells, macrophages, neutrophils), neural/glial cells, and satellite cells [22, 23]. In addition, using scRNA-seq, Ziye Xu et al. uncovered cell and lipid dynamics of fat infiltration in skeletal muscle [24]. Haibin Xi et al. profiled human skeletal muscle tissues from embryonic, fetal, and postnatal stages and constructed a “roadmap” of human skeletal muscle ontogeny across development [25]. However, our knowledge about muscle ontogeny in pigs is limited. Here, to investigate the upstream regulatory networks in myogenesis that lead to establishment of myogenic lineage and subsequent differentiation, we performed scRNA-seq and scATAC-seq of pig somite and myotome cells from Tibetan pigs (ZZ) and Duroc×Tibetan pigs (DZ) at several embryonic stages (E16, E18, E21, and E28). We produced a classification of developing myogenic cells, observed dynamic changes in gene expression and chromatin accessibility along the myogenic differentiation trajectory, and defined cell-type-specific regulatory networks. We also investigated key TFs and cell–cell interactions associated with embryonic myogenesis. Finally, the molecular and cellular impacts of early growth response 1 (*EGR1*) and Ras homolog family member B (*RHOB*) were substantiated by overexpressing the two genes in pig primary myogenic cells (PPMCs) and C2C12 myoblasts, which resulted in a promotion of myogenic differentiation. This extensive analysis enhances our understanding of the molecular and cellular dynamics in pig embryonic myogenesis and provides an invaluable resource for studying animal skeletal muscle ontogeny and human muscle diseases.

## Results

### scRNA-seq identified the major cell types in developing pig somites

**Fig. 1:**
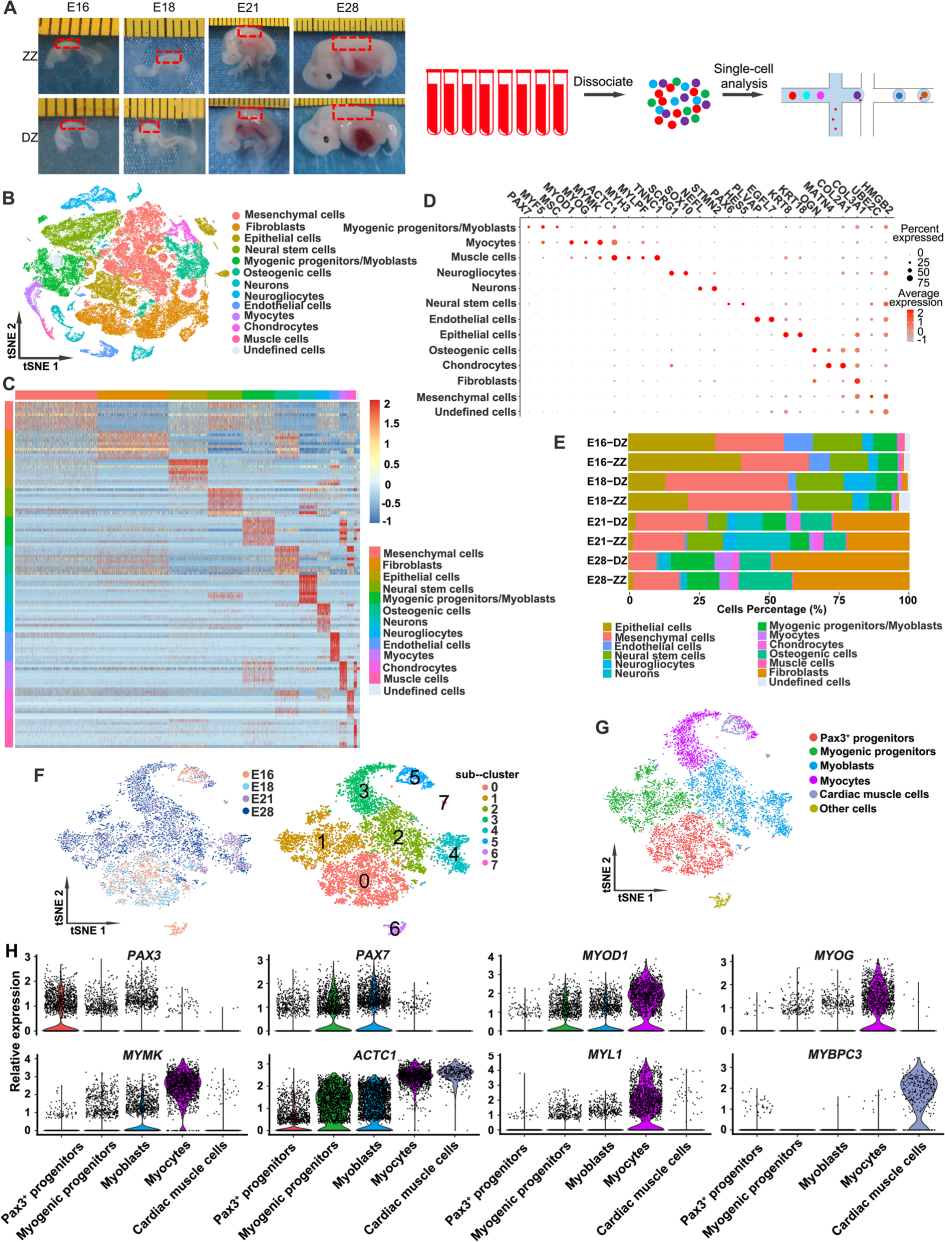
scRNA-seq identified major cell types in developing pig somites. **A** Experimental workflow schematic. Somite tissues [a mixture of embryos (*n*=5) from the same sow] of ZZ and DZ at E16, E18, E21, and myotome tissues [a mixture of embryos (*n*=5) from the same sow] at E28 were isolated. Tissue samples were dissociated into a single-cell solution and then single-cell transcriptomes were captured and analyzed using 10×Genomics. The minimum scale of the ruler in the embryo photograph is 1 mm. **B** t-Stochastic neighbor embedding (tSNE) plots showing the distribution of the main cell populations using the scRNA-seq. Using marker genes, cells were annotated as mesenchymal cells, fibroblasts, epithelial cells, neural stem cells, myogenic progenitors/myoblasts, osteogenic cells, neurons, neurogliocytes, endothelial cells, myocytes, chondrocytes, or muscle cells. Colors indicate cell types. Each dot represents one cell. **C** Heatmap showing the top 20 markers for each of the 12 cell populations. **D** Dot plot of the mean expression of canonical marker genes for 12 cell populations. **E** Bar plot showing the percentage of different cell types within each sample. **F** Visualization of myogenic cell (including myogenic progenitors/myoblasts, myocytes, and muscle cells in Fig. 1B) sub-clusters via t-SNE by developmental stage (left) and sub-cluster number (right). **G** tSNE plots showing the cell identities of myogenic cell sub-clusters. **H** Violin plots showing feature gene expression in each cell sub-cluster. Colors represent sub-clusters described in Fig. 1G

To gain a comprehensive view of the cell populations present during pig skeletal muscle ontogeny, we used scRNA-seq to evaluate somite and myotome tissues of ZZ and DZ from embryonic day (E) 16 to E28, which covers the transition from Pax3^+^ progenitors to myocytes. Single cells from 8 samples (E16-ZZ, E16-DZ, E18-ZZ, E18-DZ, E21-ZZ, E21-DZ, E28-ZZ, and E28-DZ) were processed for scRNA-seq using a Chromium system (10× Genomics) (Fig. 1A). Overall, 70,201 cells passed quality control (QC) with an average of 1892 genes per cell and 5276 unique molecular identifiers (UMIs) per cell (Additional file 1: Figure S1A, B). To exclude technical batch effects, the datasets from all samples and tissues were merged using autoencoders (AEs) and applied the batch-balanced k nearest neighbors (BBKNN) approach [26] to the latent space [27] (Additional file 1: Figure S1C). Dimensional reduction and unsupervised clustering for all 70,201 cells identified 31 distinct clusters (Additional file 1: Figure S1D). Based on differential expression analysis and the expression of selected marker genes from the literature, we manually annotated 12 distinct populations (Additional file 2: Table S1 and Fig. 1B, C), including mesenchymal cells, fibroblasts, epithelial cells, neural stem cells, myogenic progenitors/myoblasts, osteogenic cells, neurons, neurogliocytes, endothelial cells, myocytes, chondrocytes, and muscle cells. Bubble plots of marker gene expression demonstrated the accuracy of the cell annotations (Fig. 1D). Gene Ontology (GO) analysis of the differentially expressed genes (DEGs) for each cell type verified the characteristics and functions of different cells (Additional file 3: Table S2 and Additional file 1: Figure S2). We next quantified changes in the cell type percentages during somite development. As shown in Fig. 1E, the patterns of the cell populations vary considerably across development stages, with the proportions of low differentiation cells, such as epithelial cells, endothelial cells, and neural stem cells, decreased with development, while the percentages of highly differentiated cells, such as fibroblasts, osteogenic cells, and myocytes, increased gradually with development, suggesting a rapidly somite development during E16 and E28.

To further dissect the cellular heterogeneity and transcriptional landscape of developing myogenic cells, we extracted myogenic progenitors/myoblasts, myocytes, and muscle cells for further clustering. The myogenic cells were further divided into 8 distinct sub-clusters with increased resolution and annotated as Pax3^+^ progenitors, myogenic progenitors, myoblasts, myocytes, cardiac muscle cells, and other cells (Fig. 1F, G). Among them, Pax3^+^ progenitors were characterized by the highest expression level of *Pax3*, whereas myogenic progenitors and myoblasts were characterized by the expression of the muscle stem cell marker *Pax7* and the myogenic regulatory factor *MyoD*. Both myocytes and cardiomyocytes expressed the muscle cell marker *ACTC1*, but the differentially expressed skeletal myogenic cell markers *MYOG* and *MYL1* as well as the cardiomyocyte-specific marker gene *MYBPC3* [28] accurately distinguished the two cell types (Fig. 1H).

### Reconstruction of the myogenic differentiation trajectory of Pax3^+^ progenitors

**Fig. 2:**
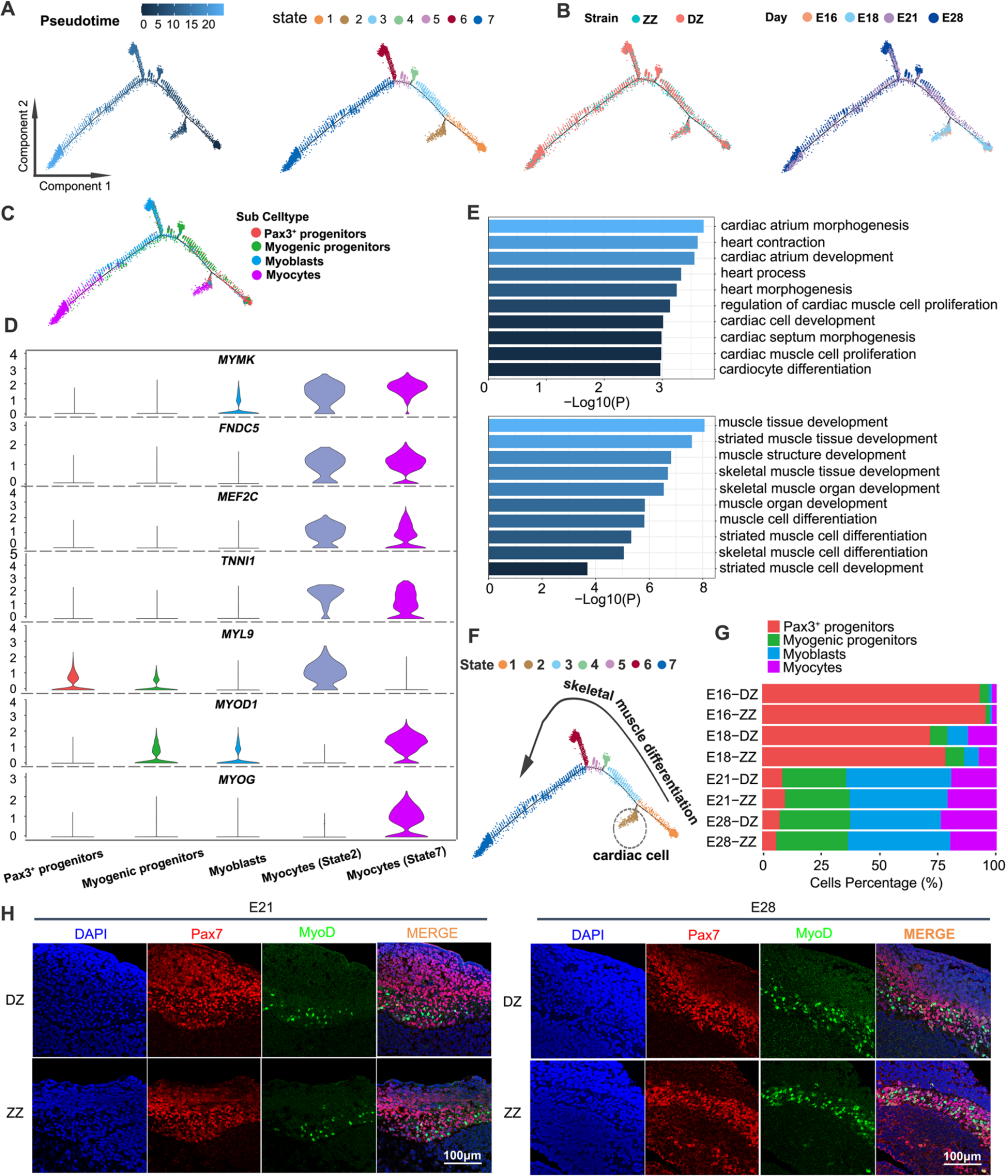
Reconstruction of the myogenic differentiation trajectory in a pseudotime manner. **A** Pseudotime analysis of myogenic cells (including Pax3+ progenitors, myogenic progenitors, myoblasts, and myocytes in Fig. 1E) was performed by Monocle 2 and revealed seven different cell states (states 1~7). The distributions of cell states were presented along with pseudotime flows. Each dot is a cell. **B** Visualization of myogenic differentiation trajectory by cell origins (left) and developmental stages (right). **C** Visualization of myogenic differentiation trajectory by cell identity. **D** Violin plots showing feature gene expression in each cell cluster. *MYMK*, *FNDC*5, *MEF2C*, and *TNNI1* are muscle development-related genes. *MYL9* is a cardiomyocyte-specific marker. *MYOD1* and *MYOG* are skeletal muscle cell-specific markers. **E** Gene Ontology (GO) analysis of the differentially expressed genes with high levels in myocytes (state 2) and myocytes (state 7). **F** Visualization of myogenic differentiation trajectory by cell state, with cardiac cells distinguished from differentiating skeletal muscle cells. **G** Bar plot showing the percentage of cells within each sample assigned to the annotated myogenic cell types. **H** Immunofluorescence staining for Pax7 and MyoD on somite cross sections of ZZ and DZ at E21 and E28. Scale bar = 100μm

To investigate the molecular processes underlying skeletal muscle development, the cells were ordered in a pseudotime manner using Monocle 2 [29]. Pseudotime trajectory analysis revealed seven different cell states (states 1~7) and presented the distributions of cell states along with pseudotime flows (Fig. 2A). An organized, branched progression of cells from Pax3^+^ progenitors to differentiated myocytes was shown by labeling individual cells using the cell population annotations from the unified atlas in Fig. 1E (Fig. 2B, C). Unexpectedly, the first small branch (state 2) was almost entirely enriched by myocytes, and the cells at the end of pseudotime trajectory (state 7) also belonged to myocytes (Fig. 2A, C). The differential gene expression analysis indicated that many muscle development-related genes were highly expressed in both states of myocytes (e.g., *MYMK*, *FNDC5*, *MEF2C*, and *TNNI1*). However, the myocytes in state 2 expressed the cardiomyocyte-specific marker *MYL9*, while those in state 7 expressed the skeletal muscle cell-specific markers *MYOD1* and *MYOG* (Fig. 2D). In addition, the DEGs with high levels in state 2 were largely involved in the regulation of biological processes such as “heart process” and “cardiac cell development,” while the DEGs with highly levels in state 7 were involved in “skeletal muscle tissue development” and “skeletal muscle cell differentiation” (Fig. 2E). These results indicated that the myocytes in state 2 were actually cardiomyocytes which were excluded from subsequent analysis (Fig. 2F).

Then, the percentages of Pax3^+^ progenitors, myogenic progenitors, myoblasts, and myocytes in each sample were calculated to assess myogenesis progression at different stages. Although the proportion of total myogenic cells in all cells of somite tissue did not change obviously (Fig. 1E), the percentages of four types of myogenic cells in different developmental stages changed significantly during E16~E28. At E16, there were almost no myoblasts and myocytes in the somites, with pax3^+^ progenitors accounting for about 90% of the four types of cells. At E18, some progenitors were committed to become myoblasts and further differentiated into myocytes. Subsequently, the proportion of pax3^+^ progenitors decreased markedly, and myogenic progenitors, myoblasts, and myocytes accounted for about 90% of the four types of cells at E21 and E28 (Fig. 2G). Dual immunostaining of Pax7 and MyoD showed that MyoD^+^ cells appeared at E21 and increased significantly at E28, indicating that E18~28 was a critical period for the establishment of skeletal muscle lineage (Fig. 2H). As expected, cells from E16 and E18 embryos tended to be distributed at the root of the trajectory suggesting versatile progenitor properties, whereas those from E21 and E28 embryos were distributed in the later part of the trajectory indicating the decreased proportion of progenitor cells and increased proportion of differentiating myogenic cells during skeletal muscle development (Fig. 2B).

In addition to differentiation, proliferation is also a major biological event during early skeletal muscle development. The cell cycle phase of each cell was predicted to evaluate the proportion of proliferating cells at different states of myogenic progression. We observed a shift in the transcriptomically defined cell cycle state accompanying the change in cell type representation (Fig. 2C and Additional file 1: Figure S3A). In the early part (states 1, 3, and 4) of the pseudotime trajectory, most of the cells are progenitors, so they are primarily predicted to be proliferating (S and G2M phases), with only a small fraction being predicted to be in G1 phase (non-proliferating). However, in the middle stage of the trajectory (State 5), proliferating cells decreased to 73.9%, while non-proliferating cells increased significantly to 26.1%. At the end of the trajectory (States 6 and 7), most of the cells have differentiated into myocytes that no longer proliferate, so they are primarily in G1 phase (75.3–85.1%) (Additional file 1: Figure S3B). Consistently, the variation in the expression of cell cycle-related genes and myogenic genes suggested that proliferating cells decreased with the differentiation trajectory (Additional file 1: Figure S3C, D).

### Transcriptome dynamics of Pax3^+^ progenitor differentiation

**Fig. 3:**
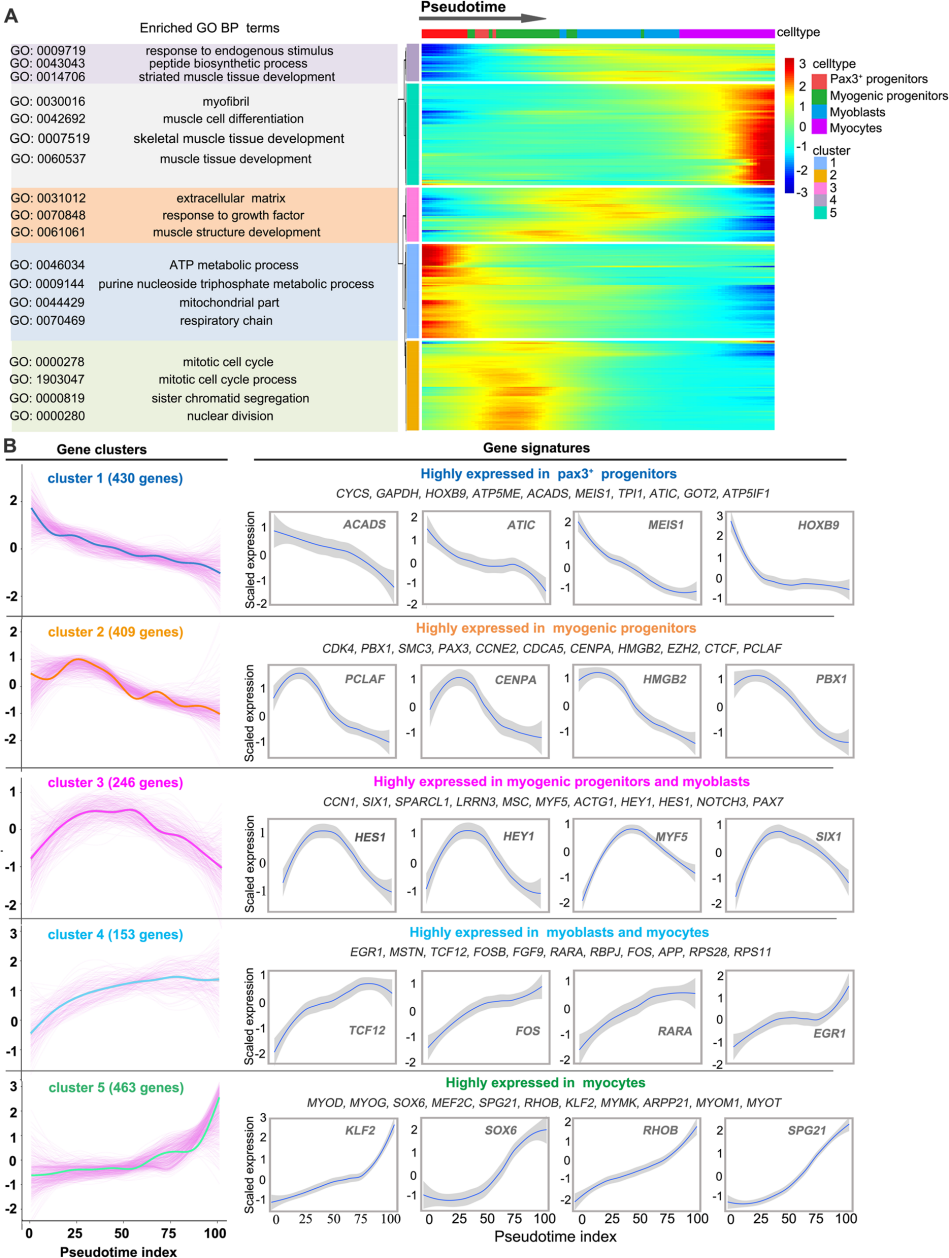
Transcriptome dynamics of the myogenic differentiation. **A** Heatmap showing the expression changes of the 1700 top differentially expressed genes (DEGs) in a pseudotemporal order, with the DEGs, were cataloged into 5 five major clusters in characterized patterns (right). The GO analysis was performed for each gene cluster, and the representative enriched biological process (BP) terms are presented (left). **B** The expression dynamics of DEGs in gene clusters. Thick lines indicate the average gene expression patterns in each cluster (left). Gene signatures and expression dynamics of representative genes in each gene cluster (right)

To gain insights into the gene expression dynamics along the trajectory, the expression changes of the 1700 top DEGs among the four types of myogenic cells (including Pax3^+^ progenitors, myogenic progenitors, myoblasts, and myocytes) were analyzed and clustered into five major categories of transcriptional gene clusters (Additional file 4: Table S3 and Fig. 3A). Genes in cluster 1, with the highest expression in Pax3^+^ progenitors, were gradually downregulated from the beginning of programming and were largely involved in the regulation of biological processes such as “ATP metabolic process” (e.g., *ACADS*, *ATIC*, and *MEIS1*) (Fig. 3B). Subsequently, gene clusters 2 and 3 were transiently upregulated but finally downregulated, representing two temporary transcriptional waves. Cluster 2 genes highly expressed in myogenic progenitors were involved in “mitotic cell cycle process” (e.g., *PCLAF*, *CENPA*, and *HMGB2*) indicating the strongest proliferation capacity of myogenic progenitors during myogenesis. Cluster 3 genes highly expressed in myogenic progenitors and myoblasts are involved in “extracellular matrix” and “response to growth factor” (e.g., *HES1*, *HEY1*, and *SIX1*) (Fig. 3B). Concurrent with cluster 3 activation, cluster 4 genes highly expressed in myoblasts and myocytes were upregulated and maintained at high expression levels until the final stage with enrichment of the GO terms “response to endogenous stimulus” and “striated muscle tissue development” (e.g., *TCF12*, *FOS*, and *EGR1*). Finally, cluster 5 genes were activated at the end of the trajectory with predominant involvement in the GO term “muscle cell differentiation” (e.g., *KLF2*, *RHOB*, and *SOX6*) (Fig. 3B). These data illustrated the trajectory of myogenic differentiation and revealed the ordered activation of transcriptional waves throughout this process.

### Single-cell chromatin accessibility profiling of pig skeletal muscle ontogeny

**Fig. 4:**
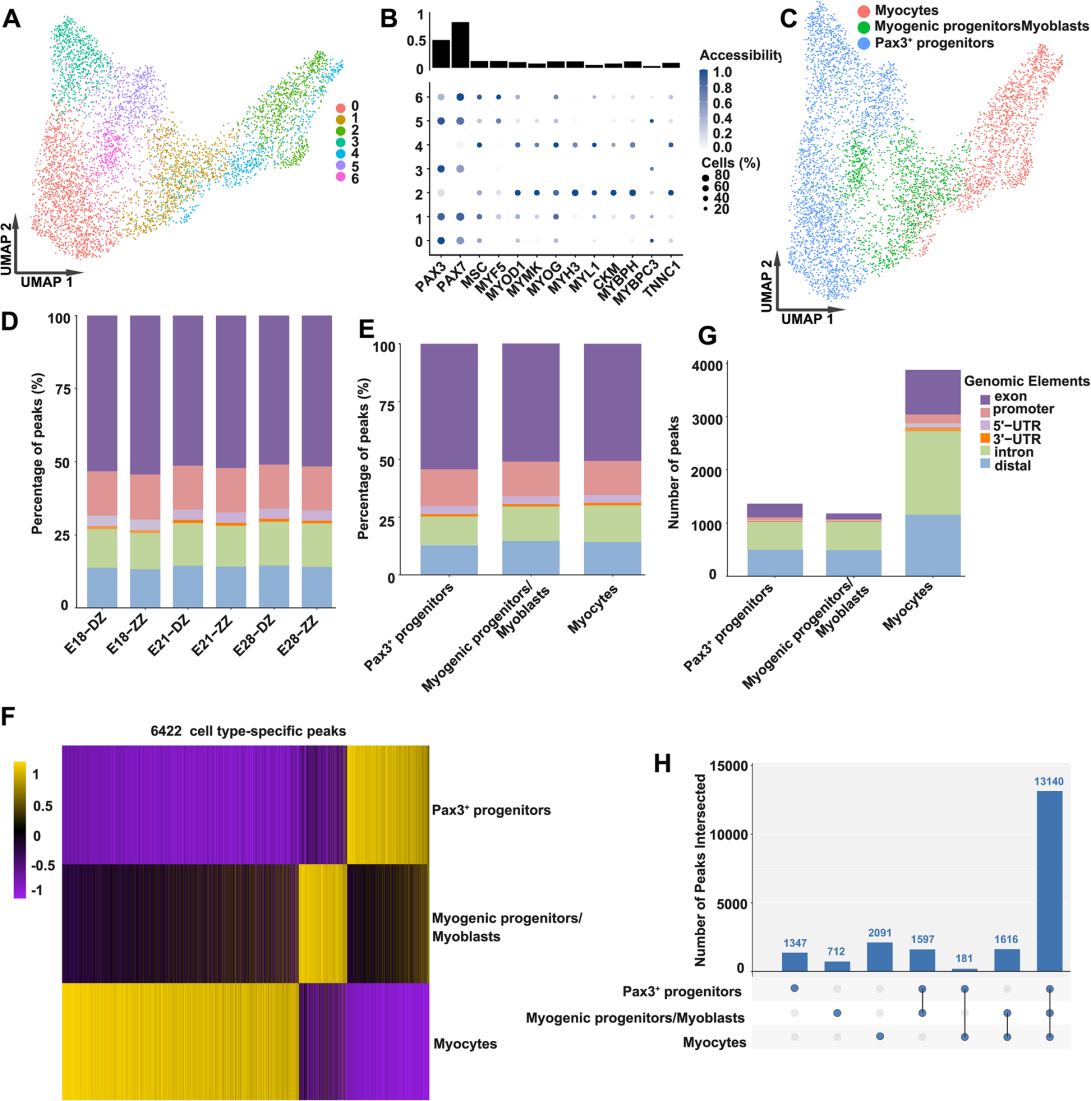
Single-cell chromatin accessibility analysis of pig myogenic cells. **A** The myogenic cells in the scATAC-seq dataset are shown in the Uniform Manifold Approximation and Projection (UMAP) space, colored by cluster. **B** Top: Bar plot showing the average accessibility of 13 selected marker genes from our scRNA-seq data considering all myogenic cells. Bottom: dot plot of the standardized accessibility of the marker genes (gene body ± 2 kb) in each of the seven clusters. For each gene, the minimum accessibility value is subtracted, and the result is divided by its maximum accessibility value. The dot size indicates the percentage of cells in each cluster in which the gene of interest is accessible. The standardized accessibility level is indicated by color intensity. **C** UMAP visualization of the myogenic cells in the scATAC-seq dataset, colored by cell identity. **D** Percentage distribution of open chromatin elements in each scATAC-seq sample. **E** Percentage distribution of open chromatin elements in scATAC-seq myogenic cell types. **F** Heatmap showing cell type-specific differentially accessible peaks (DAPs) (yellow: open chromatin, purple: closed chromatin). **G** Distribution of open chromatin elements among DAPs in myogenic cell types. **H** Number of shared and unique peaks among snATAC-seq cell types

To further investigate the regulatory events in developing myogenic cells, the single-cell chromatin accessibility landscape was analyzed (Additional file 1: Figure S4A-C). Using a shared nearest neighbor (SNN) modularity optimization-based clustering algorithm, we obtained 15 distinct clusters of differentially accessible peaks (Additional file 5: Table S4 and Additional file 1: Figure S4D). Clusters 4 and 8 were annotated as myogenic cells for their high accessibility of marker genes associated with myogenic lineage (Figure. S4E, F). Then, the myogenic cells were further divided into 7 distinct sub-clusters with increased resolution (Fig. 4A).

To explore the chromatin accessibility profiles across the seven clusters, we examined the accessibility of selected marker genes from our scRNA-seq data (Fig. 4B). In clusters 2 and 4, we observed greater accessibility of marker genes associated with myocytes (e.g., *MYOG*, *MYH3*, *MYL1*, and *CKM*) and lower accessibility of genes associated with progenitor cells (e.g., *PAX3* and *PAX7*) (Additional file 6: Table S5 and Fig. 4B). In contrast, cells in clusters 0, 3, and 5 showed greater accessibility for marker genes of progenitor cells and lower accessibility for marker genes of myocytes. Clusters 1 and 6 had mixed signatures, with greater accessibility of *PAX7*, *MSC*, *MYF5*, and *MYOG*. Based on these observations, we manually annotated the seven clusters as Pax3+ progenitors, myogenic progenitors/myoblasts, and myocytes (Fig. 4C).

To characterize different genomic elements captured by scATAC-seq data, the genome was stratified into promoters, exons, 5′ and 3′ untranslated regions, introns, and distal regions using the GENCODE annotation [30] (Additional file 1: Figure S5A, B). There was little difference in the proportions of different regions between samples or cell types, with exons accounting for about 50%, promoters, introns, and distal regions accounting for about 15% each, and 5′ and 3′ untranslated regions accounting for about 5% (Fig. 4D, E). To study the open chromatin heterogeneity across cell types and developmental stages, we derived a cell type-specific chromatin accessibility landscape by conducting pairwise Fisher’s exact test for each peak between every cluster. In total, we identified 6422 differentially accessible open chromatin peaks (DAPs) across the 3 cell types, which separated the three cell types perfectly (Additional file 7: Table S6 and Fig. 4F). Among these peaks, most were in regions characterized as distal elements or introns, while relatively few (<10%) were in the promoters or 5′ and 3′ untranslated regions (Fig. 4G and Additional file 1: Figure S5C), indicating a critical role for enhancer elements in skeletal muscle development. In addition to the cell type-specific peaks, some cell type-independent open chromatin areas (present across Pax3^+^ progenitors, myogenic progenitors/myoblasts, and myocytes) also were found, likely consisting of basal housekeeping genes and regulatory elements (Fig. 4H). The overlapping peaks between Pax3^+^ progenitors and myogenic progenitors/myoblasts, and between myogenic progenitors/myoblasts and myocytes, were more than that between Pax3^+^ progenitors and Myocytes, which is consistent with their biological similarities and differentiation process (Fig. 4H). The common peaks of the three cell types were much more than those of the other groups, revealing their close lineage relationship.

### Cell type-specific gene regulatory landscape of embryonic skeletal muscle in pigs

**Fig. 5:**
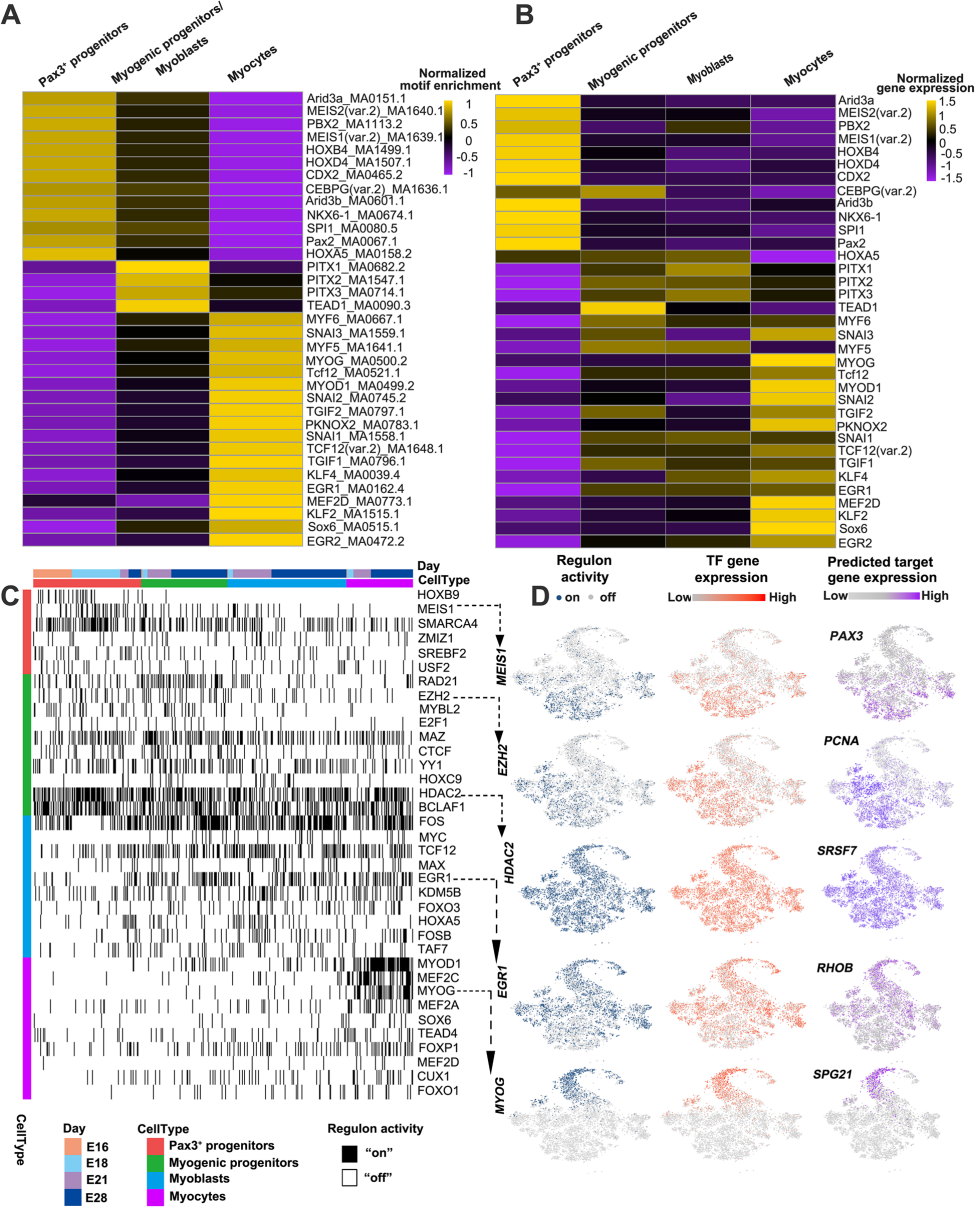
Cell type-specific gene regulatory landscape of pig embryonic skeletal muscle. **A** Heatmap showing motif enrichment analysis on the cell type-specific open chromatin regions using 10× Genomics (full results are shown in Additional file 7: Table S6). **B** TF expression *z* score heatmap that corresponding to the motif enrichment in each cell type. **C** Heatmap of cell type-specific regulons, as inferred by the SCENIC algorithm. Regulon activity was binarized to “on” (black) or “off” (white). **D** tSNE depiction of regulon activity (“on-blue”, “off-gray”), TF gene expression (red scale), and expression of predicted target genes (purple scale) of exemplary regulons for Pax3+ progenitors (*MEIS1*), myogenic progenitors (EZH2 and HDAC2), myoblasts (*EGR1*), and myocytes (*MYOG*). Examples of target gene expression of the TFs (*PAX3*, *PCNA*, SRSF7, *RHOB*, and *SPG21*) are shown in purple scale. Additional examples are given in Figure S6. The full list of regulons and their respective predicted target genes can be found in Additional file 11: Table S10

Cell type-specific chromatin opening and closing events associated with TF binding changes establish the cell type-specific regulatory landscape, resulting in cell-type specification and development. Therefore, the motif enrichment analysis was performed on the cell type-specific open chromatin regions using 10× Genomics. The full list of cell type-specific TF binding motifs is shown in Additional file 8: Table S7. We next correlated the motif enrichment with scRNA-seq TF expression (Additional file 9: Table S8). Using this combined motif enrichment and gene expression approach, the pig skeletal muscle cell type-specific TF landscape was defined (Fig. 5A, B). Correlation of RNA expression and chromatin accessibility in individual single cells revealed two characteristic patterns of ATAC–RNA pairs: (i) RNA expression of TFs directly matches accessibility of corresponding TF bindings sites as exemplified for *ARID3A*, *MEIS2*, *MEIS1*, *HOXB4*, and *HOXD4* in Pax3^+^ progenitors, and *MYOG*, *MYOD1*, *KLF2*, and *SOX6* in myocytes, suggesting that these TFs actively regulate their respective target genes at the specific developmental stage; (ii) RNA expression of TFs precedes the increase in accessibility of the corresponding TF binding sites. This scenario was apparent for *MYF6*, *MYF5*, *SNAI1*, and *TGIF1*, which reached their highest expression levels in myogenic progenitors/myoblasts but showed the strongest motif enrichment in myocytes, suggesting that additional epigenetic regulation could occur before TFs take action (Fig. 5A, B).

To study the putative target genes of TFs, single-cell regulatory network inference and clustering (SCENIC) was performed to examine TF regulon activity [31]. The activity of each regulon in each cell was quantified and then binarized to “on” or “off” based on activity distribution across cells. The SCENIC results indicated strong enrichment of *HOXB9* and *MEIS1* regulon activity in Pax3^+^ progenitors; *RAD21*, *EZH2*, and *CTCF* activity in myogenic progenitors; *TCF12*, *EGR1*, and *FOSB* activity in myoblasts; and *MYOD1*, *MEF2C*, *MYOG*, and *SOX6* activity in myocytes (Additional file 10: Table S9 and Fig. 5C). Although the activity of *MEF2*A and *MEF2D*, which belong to the same family as *MEF2C*, was elevated in myocytes, it was not as significant as *MEF2C*, indicating that they did not play a leading role in myogenesis. SCENIC also successfully inferred multiple downstream target genes. The complete list of regulons and their respective predicted target genes can be found in Additional file 11: Table S10. The scaled and binarized regulon activity is available in Additional file 12: Table S11. Examples of regulon activity, corresponding TF expression, and predicted target gene expression are depicted in Fig. 5D and Additional file 1: Figure S6A. TFs such as *SOX6*, *TEAD4*, and *FOXO1* were predicted as targets of skeletal muscle-specific TF *MYOG*, and *FOS* was predicted as a target of *FOSB*, indicating a critical transcriptional hierarchy of skeletal muscle development. Corresponding chromatin accessibility in scATAC data for these TFs and predicted target genes are shown in Figure. S6B.

### Chromatin dynamics of Pax3^+^ progenitor differentiation

**Fig. 6:**
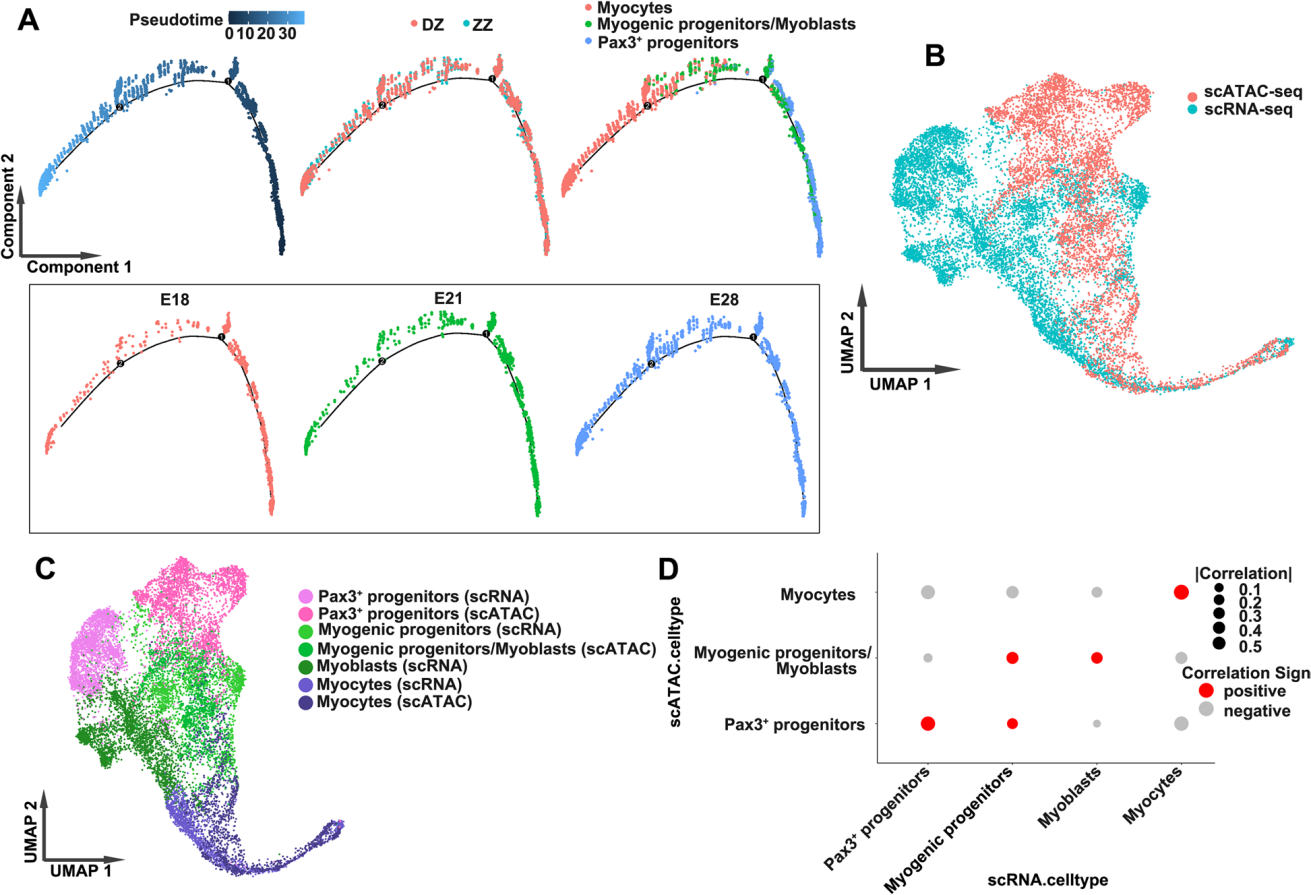
Integrated analysis of scATAC-seq and scRNA-seq data. **A** The pseudotime trajectory in the scATAC-seq dataset. **B** UMAP representation of scATAC-scRNA integration results. Cells are colored by technology (scATAC=red, scRNA=blue). **C** UMAP representation of scATAC-scRNA integration results. Cells are colored by cell type assignment. **D** Dot plot showing scATAC-scRNA integration cell type assignment using confusion matrix. Each column represents the original cell type assignment of scRNA-seq data, and each row represents the cell type assignment predicted after integration with scATAC-seq data. The size of the dots represents the absolute value of the correlation, and the red and gray dots represent the positive and negative correlations, respectively

The pseudotime trajectory in the scATAC-seq dataset was evaluated, which resulted in a similar cellular differentiation trajectory to scRNA-seq dataset (Fig. 6A). We integrated the scRNA-seq and scATAC-seq datasets to correlate and cross-validate gene expression profiles and the chromatin accessibility landscape in the myogenic cells using the Harmony algorithm [32]. The coembedded Uniform Manifold Approximation and Projection (UMAP) plots with cell type assignment for the scATAC-seq and scRNA-seq data suggested that the changes in chromatin accessibility and corresponding gene transcript expression in most myocytes occurred in a synchronous manner, whereas in other cell types, it was not fully synchronized, suggesting that other regulation is involved (Fig. 6B, C). Correlations between cell types of scATAC-seq and scRNA-seq cell types were computed with scRNA-seq variable genes (Fig. 6D). These results obtained from two independent approaches demonstrate that our two datasets are highly concordant and cross-validated.

**Fig. 7:**
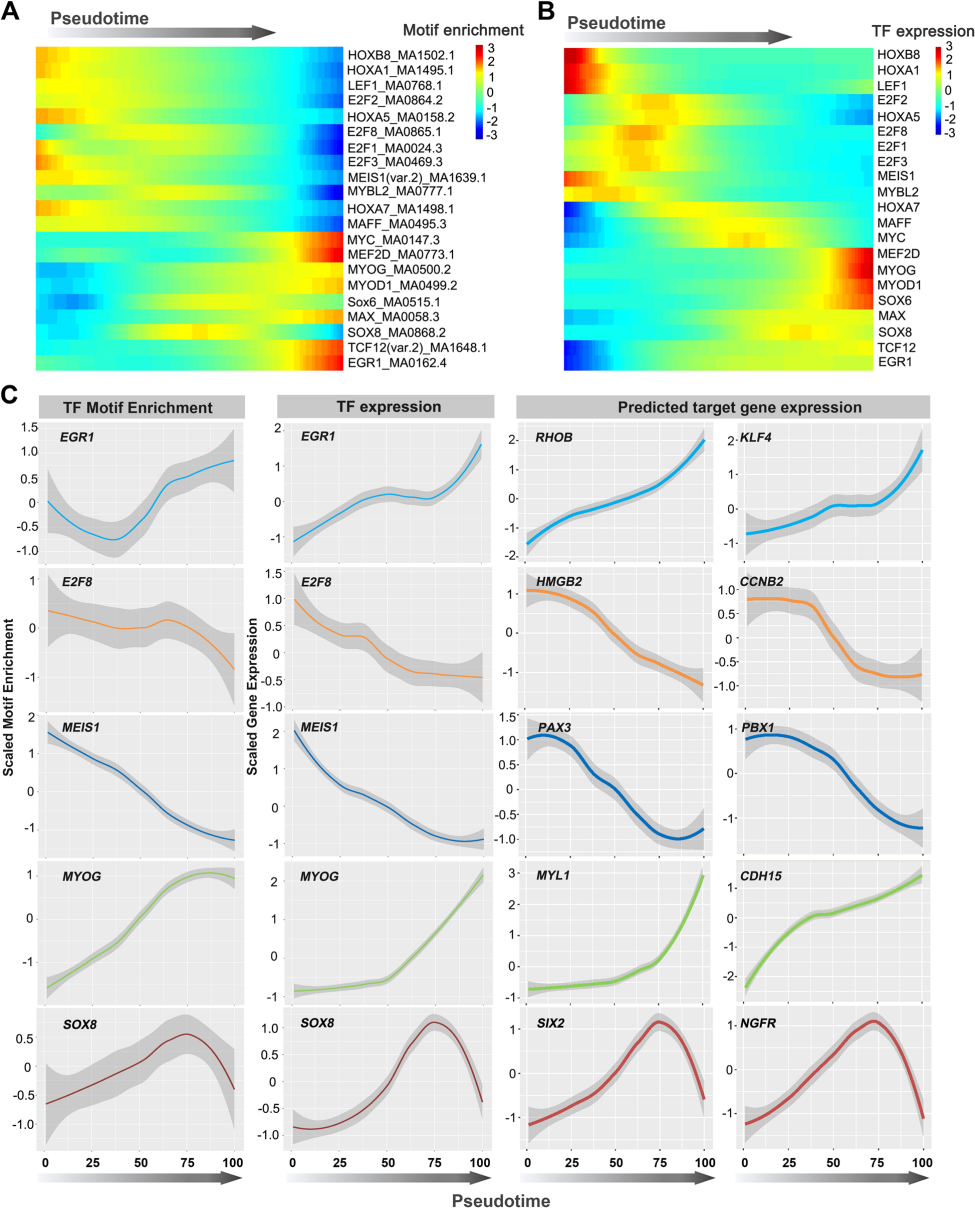
Activity and RNA expression dynamics of TFs along the pseudotime trajectory. **A** Heatmap showing the activity of TFs along the differentiation trajectory. **B** TF expression heatmap corresponding to the motif enrichment along the differentiation trajectory. **C** Pseudotime-dependent chromatin accessibility and gene expression changes along the myogenic lineages. The first column shows the dynamics of the 10× Genomics TF enrichment score. The second column shows the dynamics of TF gene expression values, and the third and fourth columns represent the dynamics of SCENIC-reported target gene expression values of corresponding TFs. Error bars denote 95% confidence intervals of local polynomial regression fitting. Additional examples are given in Additional file 1: Figure S7

We next performed pseudotime ordering of the chromatin accessibility-based TF motif enrichment of individual cells and correlated changes of the TF motif patterns with TF expression (Fig. 7A, B). To this end, we also investigated TFs and target genes differentially expressed over scRNA-seq pseudotime. We noticed good concordance of time-dependent changes in TF and predicted target gene expression along with motif enrichment, suggesting that a set of TFs cooperatively regulates myogenic differentiation (Fig. 7C and Additional file 1: Figure S7).

### EGR1 and RHOB play critical roles in myogenesis

**Fig. 8:**
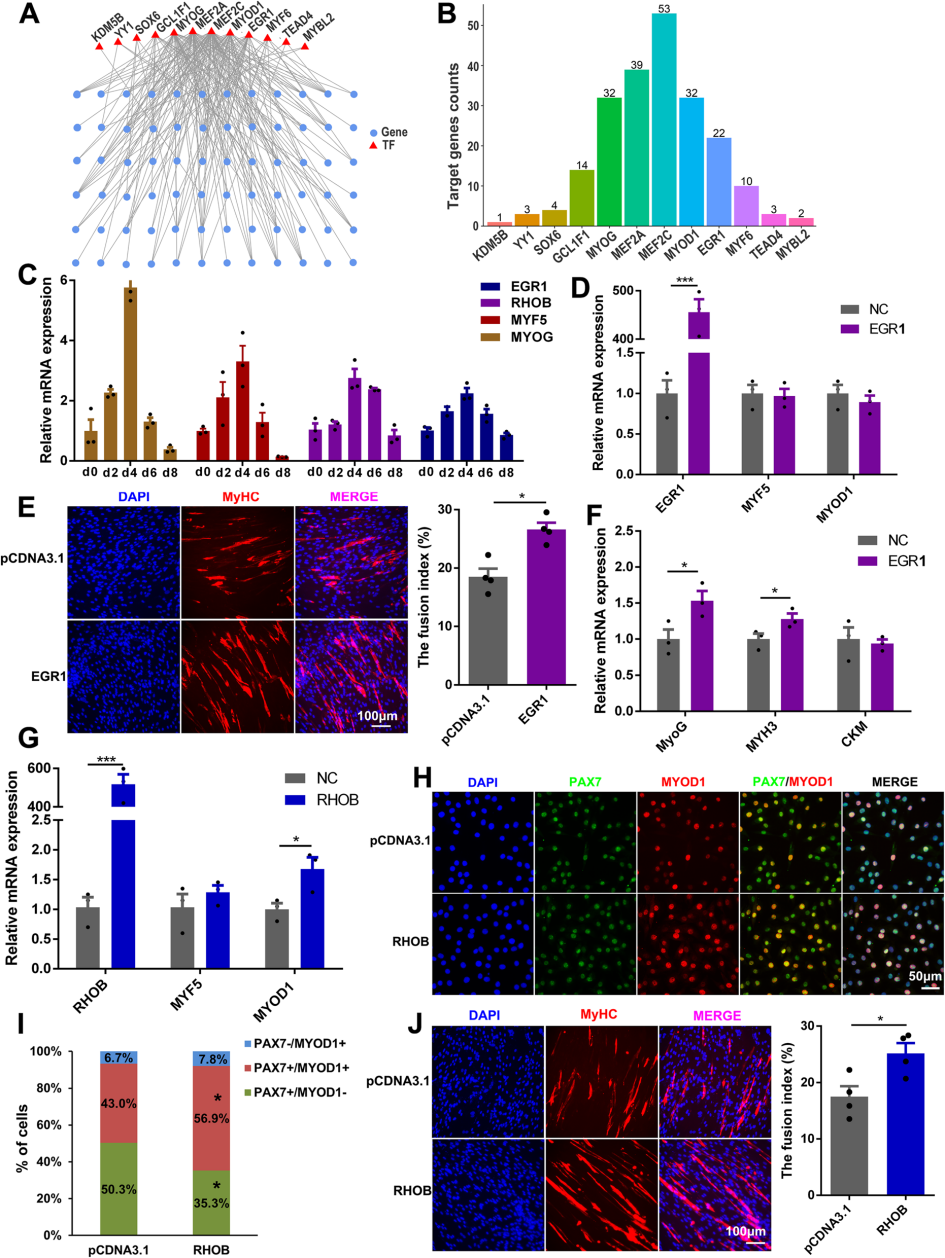
Functional analysis of *EGR1* and *RHOB* in myogenesis. **A** To explore the connection network between TFs and targets in skeletal muscle development, 172 genes associated with muscle development (http://wiki.geneontology.org/index.php/Muscle biology) were extracted as target genes. Then, 215 high-confidence annotation TF-target pairs were selected from regulon activity network to construct the regulatory network. **B** Target genes counts of TFs. **C** The mRNA levels of *EGR1*, *RHOB*, and myogenic markers during the differentiation of pig primary myogenic cells (PPMCs) at several indicated time points. When the cells were cultured in growth medium (GM) at sub-confluent density, it was defined as day 0 (day 0); when the cells reached 100% confluence, GM was changed to differentiation medium (DM). **D** The mRNA levels of *EGR1*, *Myf5*, and *MyoD* in proliferating C2C12 cells at 36 h after transfection with the pcDNA3.1-EGR1 vector. **E** Immunofluorescence staining for MyHC in PPMCs after transfection with the pcDNA3.1-EGR1 vector and differentiation induction for 5 days. The fusion index (the percentage of nuclei in fused myotubes out of the total nuclei) was calculated. **F** The mRNA levels of myogenic differentiation markers in C2C12 cells after transfection with the pcDNA3.1-EGR1 vector and induction of differentiation for 3 days. **G** The mRNA levels of *RHOB*, *Myf5*, and *MyoD*1 in proliferating C2C12 cells at 36 h after transfection with the pcDNA3.1-RHOB vector. **H** Immunofluorescence co-staining for Pax7 and MyoD in C2C12 cells transfected with the pcDNA3.1-EGR1 vector and cultured in growth medium for 36 h. **I** Statistical analysis was performed to quantify the percentages of the three myogenic cell populations. **J** Immunofluorescence staining for MyHC in PPMCs after transfection with the pcDNA3.1-RHOB vector and induced differentiation for 5 days. The fusion index was calculated. *P < 0.05, **P < 0.01, ***P < 0.001

Although the roles of several identified TFs in myoblast specification and differentiation have been established, the expression and functions of many other genes that present specific expression profiles over scRNA-seq pseudotime have not been well studied. In consideration of the possibility that pig is used as a model of muscle disease in the future, and in order to identify the conserved functional genes related to muscle development among mammals, we compared the expression of these genes in skeletal muscle from wild-type and Duchenne muscular dystrophy (DMD) mice using an RNA-Seq dataset (GSE162455). Consistent with the patterns of classical myogenic genes, most of the genes upregulated along the pseudotime trajectory were expressed at higher levels in DMD mice than that in wild-type mice (Additional file 4: Table S3 and Additional file 1: Figure S8A, B), suggesting that these genes may be induced by muscle regeneration in muscular dystrophy mice and play an important role in myogenesis. The expression levels of genes downregulated along the pseudotime trajectory were also downregulated in DMD mice, suggesting that they are involved in muscular dystrophy and may not contribute to terminal myoblast differentiation (Additional file 4: Table S3 and Additional file 1: Figure S8C). The scRNA analysis highlighted that the expression of *EGR1* and its predicted target gene *RHOB* gradually increased along the pseudotime trajectory, and scATAC analysis showed that *EGR1* reached its strongest motif enrichment in myocytes (Fig. 7C). The peaks in the vicinity of *EGR1* and *RHOB*, which were located at +3979 bp and +11,950 bp of the *EGR1* and *RHOB* transcriptional start sites (TSSs) respectively, were the most accessible in myocytes (Additional file 1: Figure S9). Based on gene expression correlation and binding motif analysis, we identified a total of 215 high-confidence annotated TF-target pairs between 12 TFs and 75 target genes, and constructed a putative gene regulatory network associated with skeletal muscle development (Fig. 8A). Among them, the number of links between transcription factor EGR1 and myogenesis-related target genes is second only to the classical myogenic transcription factor MYOD1 (Fig. 8B). RHOB is an important regulator of cell and tissue morphology and function, acting mainly through the cellular cytoskeleton [33, 34]. A few studies have revealed that RHOB is a key mediator during diverse cellular and physiological processes like cell division, cell migration in smooth muscle cells [35, 36]. Totally, we speculated that EGR1 and RHOB are likely to play positive roles during myogenic differentiation in pigs, so they were selected for in vitro functional validation.

To confirm the dynamic expression of *EGR1* and *RHOB* during myogenic differentiation, quantitative PCR (qPCR) was performed on porcine primary myogenic cells (PPMCs) at several differentiation points (day 0, day 2, day 4, day 6, and day 8). It was found that EGR1 and RHOB had the same expression pattern as the well-known myogenic differentiation makers (Fig. 8C). To validate the effect of *EGR1* and *RHOB* on myogenesis progression, they were overexpressed in PPMCs and mouse C2C12 myoblasts. Ethynyl-2′-deoxyuridine (EdU) incorporation and immunofluorescence assays showed that *EGR1* overexpression did not influence cell proliferation but promoted myogenic differentiation, inducing a significant increase in the fusion index (Fig. 8D, E, and Additional file 1: Figure S10). In line with this, the expression of myogenic differentiation markers increased when *EGR1* was overexpressed (Fig. 8F). Consistent with *EGR1*, *RHOB* overexpression did not change the proliferation ability of myoblasts, but the expression level of *MYOD1* was significantly upregulated (Additional file 1: Figure S11A-C, and Fig. 8G). This prompted us to further verify whether *RHOB* regulates myoblast fate commitment. Immunofluorescence co-staining of PAX7 and MYOD showed that *RHOB* accelerated the transformation of PAX7^+^/MYOD^−^ myogenic progenitors into PAX7^+^/MYOD^+^ myoblasts (Fig. 8H, I). After induction of differentiation, the *RHOB* overexpression group formed more myotubes with a significantly increased the fusion index (Fig. 8J, and Additional file 1: Figure S11D). These findings revealed that *EGR1* and *RHOB* are critical regulators of pig embryonic myogenesis.

### Cell–cell communications

**Fig. 9:**
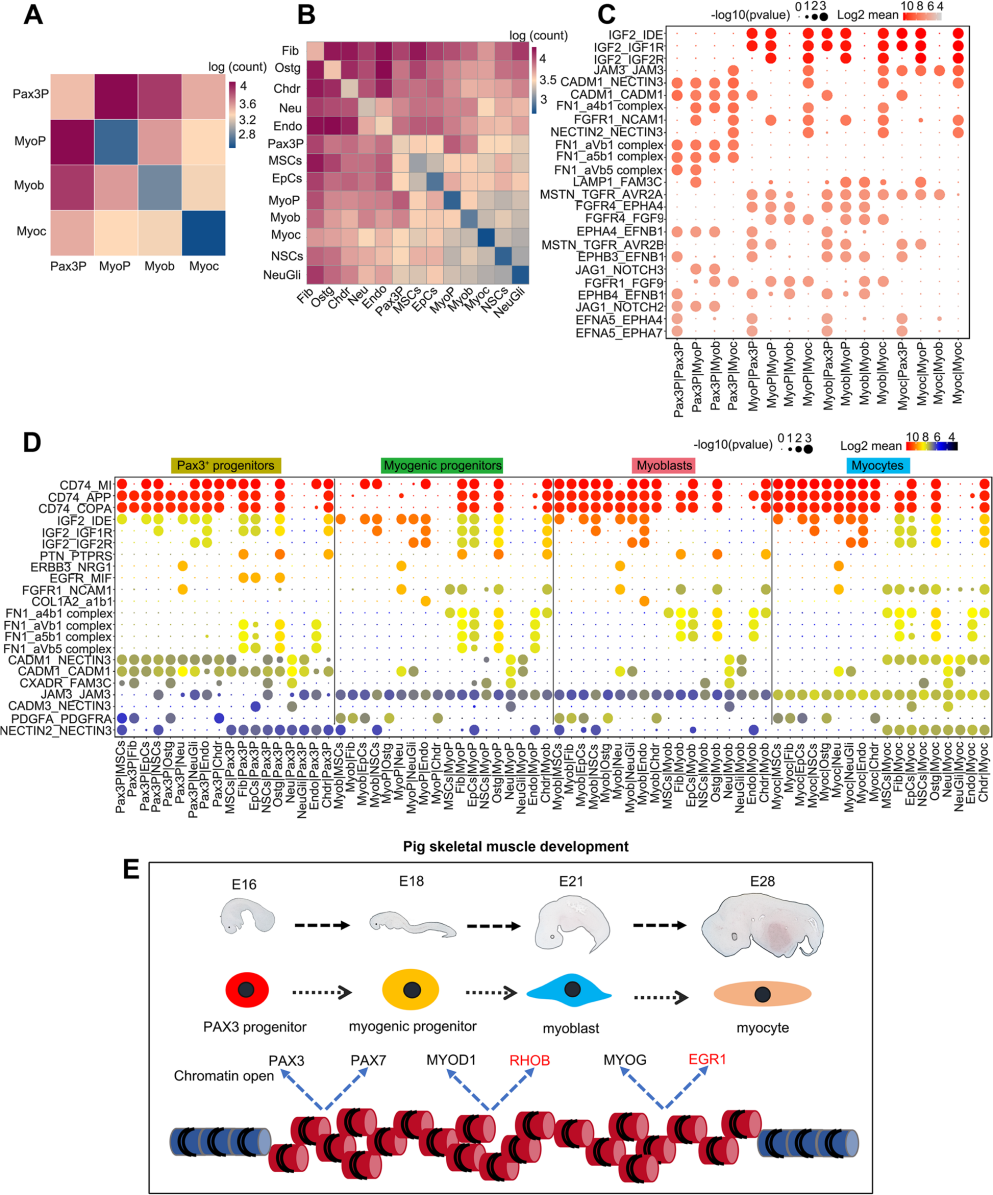
Cell–cell communication analysis in the developing pig somite. **A**, **B** Heatmaps showing the number of cell**–**cell interactions in the scRNA-seq dataset of myogenic cells (**A**) and all somite cells (**B**), as inferred by CellPhoneDB. Dark blue and dark red colors denote low and high numbers of cell**–**cell interactions, respectively. **C** CellPhoneDB-derived measures of cell**–**cell interaction scores and *p* values among myogenic cells. Each row shows a ligand**–**receptor pair, and each column shows the two interacting cell types binned by cell type. Columns are sub-ordered by first interacting cell type into Pax3+ progenitors, myogenic progenitors, myoblasts, and myocytes. The color scale denotes the mean values for all the interacting partners, where the mean value refers to the total mean of the individual partner average expression values in the interacting cell-type pairs. **D** CellPhoneDB-derived measures of cell**–**cell interaction scores and *p* values between myogenic cells and non-myogenic cells. Columns are sub-ordered by interacting myogenic cell type into Pax3+ progenitors, myogenic progenitors, myoblasts, and myocytes. **E** A diagram demonstrating the regulation of pig skeletal muscle ontogeny during embryonic development. In this model, during E16 to E 28, chromatin accessibility regulates the expression of classical and newly identified myogenic-related genes, which in turn promotes myogenic lineage fate determination and further myogenic differentiation

To predict the cellular communications involved in pig skeletal muscle ontogeny, we evaluated the potential cell–cell interactions by using CellPhoneDB [37]. First, the cellular interactions between myogenic cells were analyzed, and it was found that the interactions involving Pax3^+^ progenitor were predicted to be more significant than those involving myogenic progenitor, myoblast, and myocyte (Fig. 9A). This result illustrated that the stronger the characteristics of stem cells, the greater the possibility of their interaction with other cells. Then, all somite cell populations, including mesenchymal cells, fibroblasts, epithelial cells, neural stem cells, osteogenic cells, neurons, neurogliacytes, endothelial cells, chondrocytes, and myogenic cells, were included in the analysis. Interestingly, the interaction between cells of the same cell type was weaker than that between different cell types. Myogenic cells tended to communicate with fibroblasts, osteogenic cells, chondrocytes, neurons, and endothelial cells. In myogenic cells, except for the stronger interaction between Pax3^+^ progenitors and other cells, the communication between myogenic progenitors, myoblasts, and myocytes was less (Fig. 9B). Among the identified interactions, many were related to IGF2 and the corresponding receptors IGF1R and IGF2R (Fig. 9C), which is consistent with the well-known roles of IGF signaling in skeletal muscle development [38]. The myogenic cells showed a significant ligand–receptor interaction between FGF9 and its receptors FGFR4 and FGFR1 (Fig. 9C), underscoring its critical role in myogenic differentiation [39]. The cellular interactions between myogenic cells and non-myogenic cells were mainly related to CD74, IGF2, PTN, ERBB3, FN1, and CADM1 (Fig. 9D). ERBB3-NRG1 signal plays a critical role in sustainable myogenesis by restraining myogenic progenitors from precocious differentiation [40]. Because relatively little is known about some of these genes, the significance of these putative interactions requires further investigation. For example, in skeletal muscle, PTN is upregulated during myogenesis and postsynaptic induction [41], but little is known regarding its effects on muscle formation. Interestingly, fibroblasts, osteoblasts, and chondrocytes communicate with Pax3^+^ progenitors, myogenic progenitors, and myoblasts via PTN-PTPRS interaction pair, but this cellular communication no longer exists in differentiated myocytes (Fig. 9D).

## Discussion

In the present study, we depicted the first gene expression and open chromatin maps in developing pig somites and myotomes at single-cell resolution. Using this dataset, we explored the regulatory dynamics along the myogenic differentiation trajectory and defined cell-type-specific regulatory networks. We also investigated key TFs for embryonic myogenesis and cell–cell interactions associated with skeletal muscle development. These results shed light on the upstream regulatory networks of pig skeletal muscle ontogeny.

The widespread changes and period specificity of gene expression precisely capture the transcriptional characteristics of myogenic differentiation. For example, genes involved in vital metabolic pathways, such as “ATP metabolic process” and “purine nucleoside triphosphate metabolic process”, had their highest expression levels at the beginning of the differentiation trajectory, revealing higher metabolic activity in progenitor cells [42, 43]. The high expression of genes associated with “mitotic cell cycle” and “sister chromatid segregation” in myogenic progenitors indicated that these cells had the strongest proliferation activity during myogenesis, and they expand the pool of myogenic cells for the subsequent myofiber formation [44, 45]. The inactivation of cell cycle-related genes in myoblasts and myocytes coincided with cells exiting the cell cycle and beginning to differentiate [46]. Moreover, genes associated with “myofibril” and “muscle tissue development” were over-represented at the end of the differentiation trajectory, representing the skeletal muscle terminal differentiation processes [9, 47]. These observations suggest that distinct transcriptional programs and biological processes are activated or depressed at specific stages, which provides valuable clues for understanding their functions in skeletal muscle development.

During mammalian development, differences in chromatin state coincide with cellular differentiation and reflect changes in the gene regulatory landscape [48]. Our studies revealed a similar pattern between open chromatin information and gene expression when tracked with myogenic cell differentiation. The expression levels and motif enrichment of *HOXB8* and *HOXA1*, which are known regulators of smooth muscle or extraocular muscle differentiation [49, 50], gradually decreased along the pseudotime trajectory. Myogenic differentiation correlated with increased expression of *SOX6* and *SOX8*, among which, *SOX6* has been known to play important roles in myoblast differentiation [51]. This is the first report of *SOX8* in skeletal muscle development. Numerous studies have reported that the MYC family of basic helix–loop–helix zipper (bHLHZ) transcription factors and their binding protein MAX control multiple cellular functions and are widely involved in oncogenesis [52, 53]. C-MYC inhibits myogenic differentiation by suppressing *MYOD1* expression [54]. Here, we revealed that the expression of *MYC* and *MAX* remained high in the late-pseudotime cells, and their motif enrichment increased with differentiation, suggesting that these genes also play an essential role in the formation of skeletal muscle during pig embryo development.

Integrative single-cell RNA-seq and ATAC-seq analysis also demonstrated a cell type-specific gene regulatory landscape in pig embryonic skeletal muscle. For example, motif enrichment analysis showed that the TF binding sites of TCF12 reached their highest level of accessibility in myocytes, which is consistent with the reported function of TCF12 in regulating myocyte differentiation [55]. However, the analysis of TF regulon activity with SCENIC indicated the most vigorous TCF12 regulon activity in myoblasts. This scenario was also apparent for EGR1. The phenomenon that RNA expression of TFs precedes accessibility of related TF binding sites suggests that additional epigenetic regulation might occur before TFs take action [56]. Furthermore, although individual TFs have bound to cognate motifs, this might not be a sufficient condition to initiate target gene transcription, which would lead to mismatches between RNA-seq and ATAC-seq results [57, 58]. Of course, such conditions do not apply to all genes. TFs such as MYOD1, MYOG, SOX6, and MEF2D, critical mediators of myogenesis, were indicated to reach their highest expression level, strongest motif enrichment, and most vigorous regulon activity in myocytes, which is likely responsible for their specific and vital regulatory roles in terminally differentiation.

Distal regulatory regions (DRR) mediated chromosomal interactions play an essential role in differentiating cells [59]. Distal promoter contacts are highly cell-type specific [60] and form networks of co-regulated genes correlated with their biological functions. The cis-regulatory contact upon lineage commitment is a dynamic process that includes acquiring and losing specific promoter interactions [61]. Our results revealed that most of the differential accessible peaks were in regions characterized as distal elements or introns. Genome-wide studies showed that MRFs predominantly occupied the extragenic regions, marked by acetylated histones [62, 63]. The DRR of MYOD1 were transcribed to ^DRR^RNA which contributed to establishing a cell-type-specific transcriptional circuitry by directing chromatin-remodeling events [64]. Thus, we hypothesized that intronic enhancers [65] and DRRs play an important role in regulating myogenic differentiation, consistent with mouse kidney development [58]. However, because cis-acting regulatory elements can be located in kilo-bases away from their target genes, it is challenging to identify the true functional targets of regulatory elements [66].

Comparative analysis of transcriptome dynamics during porcine myogenic differentiation with transcriptome characteristics in the muscular dystrophy mouse model suggested that the molecular regulatory network of porcine skeletal muscle development is similar to that of muscular dystrophy. Therefore, in addition to partially revealing the molecular mechanism of skeletal muscle development in porcine embryo, this study also provides clues for studying the occurrence and development of muscular dystrophy. Furthermore, our study not only provided a large amount of high-quality data but also identified a lot of new genes involved in myogenesis. EGR1, an immediate-early response zinc-finger transcription factor with various functions in numerous contexts, including the regulation of growth and differentiation [67, 68], was validated to promote myoblast differentiation. RHOB, a member of the small Rho GTPase family [69], was demonstrated to promote the fate commitment of myogenic progenitors into myoblasts, thus actively regulating myoblast differentiation.

There are significant differences in skeletal muscle development between pig breeds differing in growth rate, muscle production, muscle fiber diameter, and meat quality [70]. In this study, the results of immunohistochemistry, scRNA, and scATAC analysis did not show significant differences between ZZ and DZ in early embryonic development, despite their considerable difference in meat production. We speculate that the early embryonic skeletal muscle development of ZZ and DZ is conserved, which may be due to their close genetic relationship. Whether there are differences between purebred Tibetan and Duroc pigs in early embryonic skeletal muscle development and which stage of skeletal muscle development is responsible for the difference in meat production between ZZ and Duroc remain to be studied in the future.

## Conclusions

In summary, our study provided critical insight into the cell type-specific gene regulatory network and cell differentiation program of pig embryonic myogenesis, and screened essential genes that regulate the development of skeletal muscle (Fig. 9E), which will provide a theoretical basis for the study of pig breeding and human muscle diseases.

## Methods

### Preparation of single-cell suspensions

Somite tissues [a mixture of embryos (*n*=5) from the same sow] of ZZ and DZ at E16, E18, E21, and myotome tissues [a mixture of embryos (*n*=5) from the same sow] at E28 were isolated and transferred to RPMI 1640 medium (GIBCO, 11875093) containing 10% fetal bovine serum (HyClone, SH30070.03) on ice. Then, the tissues were washed with PBS three times and transferred to prewarmed digestion medium containing 100 μg/mL DNase I (Sigma–Aldrich, DN25) and 0.1 g/mL Collagenase I (Sigma, C2674) in RPMI 1640. The digestion reactions were shaken vigorously for 30 s and further incubated at 37°C for 30 to 40 min in an incubator with general shaking every 6 min to release cells. The released cells were passed through a 70-μm cell strainer (BD, 352350) and were collected in 15-mL tubes. Sample viability was assessed via Trypan Blue (Thermo Fisher) and an automatic cell counter (Countstar). Cells derived from embryos of different stages and from different pigs were sorted and processed independently in all experiments. Thus, each cell can be traced back to its specific embryo and tissue of origin.

### 10× Genomics single-cell RNA sequencing (scRNA-seq)

Droplet-based scRNA-seq datasets were produced using a Chromium system (10× Genomics, PN120263) following the manufacturer’s instructions. After droplet generation, samples were transferred into prechilled 8-well tube strips (Eppendorf), and reverse transcription was performed using a Veriti 96-well thermal cycler (Thermo Fisher). After reverse transcription, cDNA was recovered using the Recovery Agent provided by 10× Genomics, followed by clean-up with Silane Dynabeads (Thermo Fisher) as outlined in the user guide. Purified cDNA was amplified for 12 cycles before being cleaned up using SPRIselect beads (Beckman). Samples were diluted at a ratio of 1:4 and run on a Bioanalyzer (Agilent Technologies) to determine the cDNA concentration. cDNA libraries were prepared as outlined by the Single Cell 3′ Reagent Kit v3 user guide with appropriate modifications to the PCR cycles based on the calculated cDNA concentration (as recommended by 10× Genomics).

### scRNA-seq data analysis

#### Quality control and doublet removal

Raw sequencing data were aligned to the pig gene expression reference Sscrofa11 (the 10× reference database was prepared with the gene annotation gff3 dataset v11.1.98 and genomic fasta dataset v11.1.98, which were downloaded from Ensemble). The CellRanger (10× Genomics) analysis pipeline was used to generate a digital gene expression matrix from these data. The raw digital gene expression matrix (UMI counts per gene per cell) was filtered, normalized, and clustered using R [v3.5.2, https://www.R-project.org/]. Cell and gene filtering was performed as follows: cells with fewer than 200 genes detected, or more than 30,000 UMIs and cells containing greater than 10% of reads from mitochondrial genes were removed. To eliminate doublets, cells with more than 40,000 transcripts were identified using DoubletFinder [71] and then removed from the dataset. Genes detected (UMI count > 0) in fewer than three cells were removed. A total of 70,201 cells were included in downstream analysis, with an average of 1892.47 genes per cell and 5276.32 UMIs per cell.

#### Cell clustering and cell-type annotation

Normalization was performed in the Seurat R package (v3.1.1) using the default parameters [72]. Multiple samples were integrated by the CCA-based anchor method [73]. After principal component analysis (PCA), the first 30 principal components were selected for clustering the cells using standard package procedures. The Louvain algorithm with a resolution of 0.6 was used to cluster cells, which resulted in 31(29) distinct cell clusters. A gene was considered to be differentially expressed if it was detected in at least 10% of one group and with at least 0.25 log fold change between two groups, and a Benjamini–Hochberg (BH) adjusted *p* value < 0.05 in Wilcoxon rank-sum test was considered to indicate significance. To investigate the heterogeneity within the myogenic cells, we conducted sub-clustering analysis of myogenic cells. Using recently reported marker genes, we identified 4 myogenic sub cell types, including Pax3+ progenitors (Pax3P), myogenic progenitors (MyoP), myoblasts (Myob), and myocytes (Myoc).

#### scRNA-seq trajectory analysis

Monocle 2 (version 2.10.1) [74] was used to construct the pseudotemporal path of myogenic cell differentiation. The myogenic cells were ordered in pseudotime along a trajectory using reduceDimension with the DDRTree method and orderCells functions. We detected genes that followed similar kinetic trends along the myogenic cell trajectory from the starting state. Hierarchical clustering was applied to cluster genes into five subgroups according to the expression patterns. A gene expression heatmap following pseudotime was plotted by the plot-pseudotime-heatmap function. Gene expression curves following pseudotime were calculated and plotted according to the mean value of gene expression of each pseudotime unit. The *x*-axis (pseudotime, scaled) is the conversion value mapped from the pseudotime value of the cell in the time trajectory analysis result to the interval [0,100].

### Gene regulatory network inference

To identify TFs and characterize cell states, we employed cis-regulatory analysis using the R package SCENIC v1.1.2-2 with human orthologous genes; this program infers gene regulatory networks based on coexpression and DNA motif analysis. The network activity is then analyzed in each cell to identify recurrent cellular states. In short, TFs were identified using GENIE3 (v1.4.3) and compiled into modules (regulons), which were subsequently subjected to cis-regulatory motif analysis using RcisTarget (v1.2.1) with two gene-motif rankings: 10 kb around the TSS and 500 bp upstream. The regulon activity in every cell was then scored using AUCell (v1.4.1). Finally, binarized regulon activity was projected into t-distributed stochastic neighbor embedding (t-SNE) plots.

### Ligand–receptor interactions

To assess cellular crosstalk between different cell types, we used human orthologous genes converted UMI count data and the Python package CellPhoneDB v2.1.2 with the human database v2.0.0 to infer cell–cell communication networks from scRNA-seq data as per the authors’ instructions. Only interactions that met the *p* values threshold (*p* value < 0.05) were considered.

### Single-cell ATAC sequencing (scATAC-seq)

scATAC-seq was performed using the 10× Chromium platform. All protocols to generate scATAC-seq data, including sample preparation, library preparation, and instrument and sequencing settings, are described below and are available at https://support.10xgenomics.com/single-cell-atac.

*scATAC-seq data analysis*

#### Cell filtering and cell clustering

Raw sequencing data aligned to the pig genome ATAC reference Sscrofa11 (10× reference database was prepared with gene annotation gff3 data v11.1.98 and genomic fasta data v11.1.98 downloaded from the Ensemble). The digital peaks matrix was quantified using the Cell Ranger ATAC (10× Genomics, v1.2.0) analysis pipeline. The following analysis was performed with the Seurat R package (v3.1.1) and Signac R package (v0.2.2). We kept only those valid barcodes that passed the following thresholds: (1) number of fragments ranging from 2000 to 30,000, (2) mitochondria ratio less than 2%, (3) transcription start site (TSS) enrichment score (by TSSEnrichment function) between 2 and 40, (4) strength of the nucleosome signal per cell (by NucleosomeSignal function) less than 5, and (5) fraction of reads overlapping peaks per cell greater than 50%. After this stringent quality control, we obtained 48,514 single cells. Normalization and latent semantic indexing (LSI) dimensionality reduction were performed for each sample independently with the RunTFIDF and RunSVD functions. Batch correction by samples was applied by Signac on the first 50 LSI components. We applied the UMAP and TSNE algorithms to the first 50 LSI components corrected by Signac. We identified 15 distinct clusters using the Seurat function FindClusters (resolution equal to 0.3).

#### Genomic element stratification

Pig ssc11 genome annotation files were downloaded from Ensembl (http://ftp.ensembl.org/pub/release-104/gtf/sus_scrofa/Sus_scrofa. -Sscrofa11.1.104.gtf.gz). The 2-kb regions upstream of each TSS were included as promoter regions. We considered only open chromatin peaks detected in more than 10% of myogenic cells. Since one open chromatin region could overlap with multiple genomic elements, we defined the priority order for the genomic elements as follows: exon > promoter > 5′-UTR > 3′-UTR > intron > distal elements. For example, if one peak overlapped with both an exon and a 5′-UTR, the algorithm counted it as an exon-region peak.

#### Identification of differentially accessible peaks

Differentially accessible peaks were defined as peaks detected in at least 10% of cells in one group and exhibited a log fold change of at least 0.25 between two groups. The significance level was set as a Benjamini–Hochberg (BH) adjusted *p* value < 0.05 in the logistic regression framework by the FindMarkers function with the parameter latent.vars = 'peak_region_fragments'.

#### scATAC trajectory analysis

The imputed expression data of scATAC-seq were generated by integrating scRNA-seq and scATAC-seq data with the TransferData function. Monocle 2 was used to construct a pseudotemporal path from the imputed expression data.

#### Predict cis-regulatory elements

We implemented two methods to study cis-regulatory elements in the snATAC-seq data. The first method was direct prediction. Peaks that overlapped with the TSSs of specific genes were assigned to cis-regulatory elements to those genes. The second method was the supplementary method, which followed Miao et al. There was coenrichment of the snATAC-seq cell-type-specific peaks and scRNA-seq cell-type-specific genes in the genome. This method links a gene with a peak if (1) the gene and peak were both specific to the same cell type, (2) the gene and peak were in cis, meaning that the peak was within ±100 kb region of the TSS of the corresponding gene, and (3) the peak did not directly overlap with the TSS of the gene.

### Correlation of scATAC-seq and scRNA-seq data

The consistency of myogenic cell subtypes defined in scRNA and scATAC was evaluated with correlation analysis. Briefly, we summed the peaks intersecting the gene body and the region 2 kb upstream to calculate a gene activity score for each gene in each cell. This procedure is implemented in the Create Gene Activity Matrix in Seurat v3. Subsequently, the average gene activity score for scATAC and the average gene expression for scRNA were calculated for each cell type. The Pearson correlations of these two average values of scRNA-seq variable genes were computed for each cell type between the two data types.

### Integrated analysis of scATAC-seq and scRNA-seq data

scRNA-seq and scATAC-seq data from myogenic cells were integrated using the transfer procedure implemented in Signac v0.2.5 and Seurat v3.2.0. Briefly, the expression levels were imputed from the scATAC-seq data by defining a genomic region for each gene, which included the gene body and 2 kb upstream of the transcription start site, and taking the sum of scATAC-seq fragments within that region (genes from chromosomes X, Y, and M were excluded). Anchors were identified for scRNA- and scATAC-seq using the Find Transfer Anchors function with the canonical correlation analysis (CCA) reduction method with the scRNA-seq dataset as a reference. Then, scATAC-imputed gene expression values were projected on the scRNA expression values and weighted by LSI reduction by TransferData. Merged scATAC-seq imputed expression data and scRNA-seq data were then subjected to scaling, PCA reduction, and UMAP dimensional reduction to completely coembed the two types of data.

### Cell culture, proliferation, and differentiation

Pig primary myogenic cells (PPMCs) were maintained in our laboratory. C2C12 cells were purchased from the American Type Culture Collection (ATCC). The cells were cultured in Dulbecco’s modified Eagle’s medium (DMEM) with 10% (v/v) fetal bovine serum (growth medium, GM). When the cells were cultured in GM at sub-confluent densities, it was defined as day 0 (d 0). To induce differentiation, cells were switched to DMEM with 2% horse serum (differentiation medium, DM) after reaching 100% confluence and then maintained in the culture medium for another 2, 3, 4, 5, 6, or 8 days (day 2, day 3, day 4, day 5, day 6, or day 8). All cells were cultured in a 37°C incubator with 5% CO_2_. We examined cell proliferation using a CellLight EdU DNA Cell Proliferation Kit (RiboBio, China), as described previously [75].

### Gene overexpression

For the *EGR1* and *RHOB* expression vectors, the coding sequences (CDSs) of the pig *EGR1* and *RHOB* genes were inserted into the pcDNA3.1 vector (Invitrogen, Carlsbad, USA). Pig primary myogenic cells and C2C12 cells were seeded into 6- or 12-well plates at 12 h before treatment and then transfected with expression plasmids using Lipofectamine 3000 (Invitrogen, Carlsbad, USA). Transfections were performed in at least triplicate for each experiment.

### Quantitative real-time PCR (qRT–PCR)

Total RNA was extracted from cultured cells according to the instructions of TRIzol® Reagent (Invitrogen, Shanghai, China), and cDNA was synthesized from 1 μg of total RNA using a reverse-transcription kit (Genestar, Beijing, China). Real-time quantitative PCR (qPCR) was performed using a SYBR Green qPCR Kit (Genestar, Beijing, China) and detected on a LightCycler 480 II system (Roche, Basel, Switzerland). The primers used for qPCR are given in Additional file 13: Table S12. Gapdh was used as an internal control, and all reactions were run in triplicate.

### Immunofluorescence

Cells cultured in 12-well plates were fixed in 4% paraformaldehyde for 10 min, followed by permeabilization in 0.5% Triton X-100 for 15–20 min. The cells were blocked with 4% BSA in Tris-buffered saline with Tween (TBST) for 1 h. Then, the cells were incubated with primary antibodies overnight at 4°C. Afterwards, the cells were washed in PBS three times and incubated with secondary antibodies for 1 h at room temperature. Finally, the cells were washed three times in PBS, and the nuclei were counterstained with 4′,6-diamidino-2-phenylindole (DAPI; 1:1000 in PBS). The antibodies used were as follows: anti-MyoD antibody (Abcam, ab212662), anti-Pax7 antibody (Thermo Fisher Scientific, PA1117), anti-Fast Myosin Skeletal Heavy chain antibody (Abcam, ab51263), anti-mouse IgG (H+L), F (ab')2 fragment (Alexa Fluor 555 conjugate) (Cell Signaling, #4409), and anti-rabbit IgG(H+L), F (ab')2 fragment (Alexa Fluor 488 conjugate) (Cell Signaling, #4412). Immunostaining images were obtained via fluorescence microscopy on an inverted microscope (Nikon, Tokyo, Japan).

### Immunohistochemistry

Somite tissues of ZZ and DZ at E21 and E28 were fixed in 4% paraformaldehyde for 19 h at 4°C, then dehydrated using gradient alcohol and embedded with paraffin. Paraffin-embedded samples were cut into 5-μm somite cross sections. The paraffin sections were placed in an oven at 64°C for 30 min and immediately moved to xylene for dewaxing. The sections were rehydrated in gradient alcohol, and antigen retrieval was performed using citrate antigen retrieval solution. Finally, immunofluorescence staining was performed using IHC kit (Abcam, Cambridge, UK) according to the manufacturer’s instructions. The antibodies used were as follows: anti-MyoD antibody (Cell Signaling Technology, 13812S), anti-Pax7antibody (Abcam, ab199010), Anti-rabbit IgG (H+L), F (ab')2 Fragment (Alexa Fluor® 488 Conjugate) (CellSignaling Technology, #4412), and Anti-mouse IgG (H+L), F (ab')2 Fragment (Alexa Fluor® 555 Conjugate) (CellSignaling Technology, #4409). Immunostaining images were obtained with a fluorescence microscope (Nikon, Tokyo, Japan).

### Statistical information

The differential accessibility analysis was conducted by logistic regression. The differential expression analysis was conducted using the Wilcoxon rank sum test. Motif enrichment was determined based on the modified *z* score (https://support.10xgenomics.com/single-cell-atac/software/pipelines/latest/algorithms/overview#zscore). All statistical tests were corrected for multiple testing using the Bonferroni method, and a significance level of 0.05 was used throughout the manuscript.

## Supplementary Information

**Additional file 1: Figure S1.** Quality control and batch effect correction in scRNA-Seq, related to Figure 1 A. Violin plots showing the number of expressed genes, the number of reads uniquely mapped against the reference genome, and the fraction of mitochondrial genes compared to all genes per cell in scRNA-Seq data. B. Box plot showing the number of genes (left) and the number of uniquely mapped reads (right) per cell in each identified cell type in scRNA-Seq data. C. tSNE plot visualization of the sample source for all 70,201 cells. Each dot is a cell. Different colors represent different samples. D. tSNE plot visualization of unsupervised clustering analysis for all 70,201 cells based on scRNA-Seq data after quality control, which gave rise to 31 distinct clusters. **Figure S2.** Gene Ontology (GO) analysis of the DEGs for each cell type was performed and the representative enriched GO terms are presented, related to Figure 1. **Figure S3.** Expression of selected marker genes along the differentiation trajectory, related to Figure 2 A. tSNE plot demonstrating cell cycle regression (left). Visualization of myogenic differentiation trajectory by cell cycle phases (G1, S, and G2/M) (right). B. Donut plots showing the percentages of cells in G1, S, and G2M phase at different cell states. C. Expression levels of cell cycle-related genes in the myogenic cells organized into the Monocle trajectory. D. Expression levels of muscle related genes in the myogenic cells organized into the Monocle trajectory. **Figure S4.** Unsupervised clustering analysis for all cells in scATAC-Seq data and myogenic-specific scATAC-seq peaks, related to Figure 4 A-C. tSNE plot visualization of the sample source for all 48514 cells in scATAC-Seq. Each dot is a cell. Different colors represent different pigs (A), different embryonic stages (B), or different samples (C). D. tSNE plot visualization of unsupervised clustering analysis for all 48514 cells after quality control in scATAC-Seq data, which gave rise to 15 distinct clusters. E. tSNE plot visualization of myogenic cells and other cells. Clusters 4 and 8 in Figure S4D were annotated as myogenic cells due to their high levels of accessibility of marker genes associated with myogenic lineage. F. Genome browser view of myogenic-specific peaks at the TSS of *MyoG* and *Myf5* for myogenic cells and other cells in the scATAC-seq dataset. **Figure S5.** Percentage distribution of open chromatin elements in scATAC-Seq data, related to Figure 4 A. Distribution of open chromatin elements in each snATAC-seq sample. B. Distribution of open chromatin elements in snATAC-seq of myogenic cell types. C. Percentage distribution of open chromatin elements among DAPs in myogenic cell types. **Figure S6.** Integrative analysis of transcription factors and target genes, related to Figure 5 A. tSNE depiction of regulon activity (“on-blue”, “off-gray”), TF gene expression (red scale), and expression of predicted target genes (purple scale) of *MyoG*, *FOSB*, and *TCF12*. B. Corresponding chromatin accessibility in scATAC data for TFs and predicted target genes are depicted. **Figure S7.** Pseudotime-dependent chromatin accessibility and gene expression changes, related to Figure 7. The first column shows the dynamics of the 10× Genomics TF enrichment score. The second column shows the dynamics of TF gene expression values, and the third and fourth columns represent the dynamics of the SCENIC-reported target gene expression values of corresponding TFs, respectively. **Figure S8.** Myogenesis related gene expression in DMD (Duchenne muscular dystrophy) mice. Comparison of RNA-seq data of flexor digitorum short (FDB), extensor digitorum long (EDL), and soleus (SOL) in DMD and wild-type mice including 2- month and 5-month age. A. The expression levels of myogenesis related genes (Myod1, Myog, Myf5, Pax7). B. The expression levels of related genes that were upregulated during porcine embryonic myogenesis (EGR1, RHOB, KLF4, SOX8, NGFR, MAX, RBFOX2, ANXA6, HES6, RASSF4, PLS3, SPG21). C. The expression levels of related genes that were downregulated during porcine embryonic myogenesis COX5A, HOMER2, BNIP3, CNCS). Data were obtained from the GEO database (GSE162455; WT, *n* = 4; DMD, *n* = 7). **Figure S9.** Genome browser view of differentially accessible peaks at the TSS of *EGR1* and *RHOB* between myogenic cells in the scATAC-seq dataset, related to Figure 8. **Figure S10.** Functional analysis of *EGR1* in myogenesis, related to Figure 8 A-B. EdU assays for the proliferation of pig primary myogenic cells (A) and C2C12 myoblasts following *EGR1* overexpression. C. qPCR analysis of the mRNA levels of cell cycle regulators in C2C12 cells following *EGR1* overexpression. D. Immunofluorescence staining for MyHC in C2C12 cells following *EGR1* overexpression and differentiation for 3 d. Then, the fusion index was calculated. **Figure S11.** Functional analysis of *RHOB* in myogenesis, related to Figure 8 A-B. EdU assays for proliferation of pig primary myogenic cells (A) and C2C12 myoblasts following *RHOB* overexpression. C. qPCR analysis of the mRNA levels of cell-cycle regulators in C2C12 cells following *RHOB* overexpression. D. Immunofluorescence staining for MyHC in C2C12 cells following *RHOB* overexpression and differentiation for 3 d. Then, the fusion index was calculated.

**Additional file 2: Supplementary Table S1.** DEG between 31 distinct cell clusters in scRNA-seq data after ambient RNA cleaning. Related to Figure 1.

**Additional file 3: Supplementary Table S2.** Gene Ontology analysis of the DEGs for each cell type. Related to Figure S2.

**Additional file 4: Supplementary Table S3.** The 1,700 top DEGs among 4 myogenic cell types were analyzed and clustered into five major categories of transcriptional gene clusters according to their expression changes. Related to Figure 3.

**Additional file 5: Supplementary Table S4.** Differentially accessible peaks between 15 distinct clusters in scATAC-seq data. Related to Figure 4.

**Additional file 6: Supplementary Table S5.** Differentially accessible peaks between 7 distinct sub-clusters of myogenic cells in scATAC-seq data. Related to Figure 4.

**Additional file 7: Supplementary Table S6.** Cell type-specific open chromatin derived from scATAC-seq analysis. Related to Figure 4.

**Additional file 8: Supplementary Table S7.** Full list of cell type-specific motif enrichment. Related to Figure 5.

**Additional file 9: Supplementary Table S8.** Expression of transcription factors associated with cell type-specific motif enrichment. Related to Figure 5.

**Additional file 10: Supplementary Table S9.** The top 10 regulons and respective target genes inferred by SCENIC. Related to Figure 5.

**Additional file 11: Supplementary Table S10.** The complete list of regulons and their respective predicted target genes inferred by SCENIC. Related to Figure 5.

**Additional file 12: Supplementary Table S11.** Scaled and binarized regulon activities in each cell type inferred by SCENIC. Related to Figure 5.

**Additional file 13: Supplementary Table S12.** The primers for qPCR. Related to Figure 8, Figure S10, and Figure S11.

## Data Availability

All data generated or analyzed during this study are included in this published article, its supplementary information files and publicly available repositories. Raw data and processed data of scRNA-seq and scATAC-seq have been deposited in GEO (https://www.ncbi.nlm.nih.gov/geo/query/acc.cgi?acc=GSE206914) with the accession codes GSE206914 [76]. In this study, we downloaded public RNA-Seq dataset of skeletal muscle from wild-type and Duchenne muscular dystrophy (DMD) mice with an accession number GSE162455 (https://www.ncbi.nlm.nih.gov/geo/query/acc.cgi?acc=GSE162455) [77]. All codes used for data analysis and graph generation in this research could be obtained from https://github.com/wangxiaoyu0687/Integrative-single-cell-RNA-seq-and-ATAC-seq-analysis-demonstrates-cellular-myogenic-differentiation.

## Abbreviations

MRFs: Myogenic regulatory factors
ZZ: Tibetan pig
DZ: Duroc×Tibetan pig
scRNA-seq: Single-cell RNA sequencing
scATAC-seq: Single-cell transposase-accessible chromatin sequencing
TF: Transcription factor
EGR1: Early growth response 1
RHOB: Ras homolog family member B
PPMCs: Pig primary myogenic cells
E: Embryonic day
QC: Quality control
UMIs: Unique molecular identifiers
AEs: Autoencoders
BBKNN: The batch-balanced *k* nearest neighbors
DEGs: Differentially expressed genes
SNN: Shared nearest neighbor
DAPs: Differentially accessible open chromatin peaks
SCENIC: Single-cell regulatory network inference and clustering
UMAP: Uniform manifold approximation and projection
DMD: Duchenne muscular dystrophy
TSSs: Transcriptional start sites
qPCR: Real-time quantitative PCR
EdU: Ethynyl-2′-deoxyuridine
DRR: Distal regulatory regions
PCA: Principal component analysis
BH: Benjamini–Hochberg
t-SNE: t-distributed stochastic neighbor embedding
CCA: Canonical correlation analysis
ATCC: American Type Culture Collection
DMEM: Dulbecco’s modified Eagle’s medium
GM: Growth medium
DM: Differentiation medium
CDS: Coding sequence
TBST: Tris-buffered saline with Tween
DAPI: 4′,6-Diamidino-2-phenylindole
